# Differences in DNA methylation profile of Th1 and Th2 cytokine genes are associated with tolerance acquisition in children with IgE-mediated cow’s milk allergy

**DOI:** 10.1186/s13148-015-0070-8

**Published:** 2015-03-31

**Authors:** Roberto Berni Canani, Lorella Paparo, Rita Nocerino, Linda Cosenza, Vincenza Pezzella, Margherita Di Costanzo, Mario Capasso, Valentina Del Monaco, Valeria D’Argenio, Luigi Greco, Francesco Salvatore

**Affiliations:** Department of Translational Medical Science, University of Naples ‘Federico II’, Via S.Pansini, 5 80131 Naples, Italy; European Laboratory for the Investigation of Food-Induced Diseases, University of Naples ‘Federico II’, Via S.Pansini, 5 80131 Naples, Italy; Department of Molecular Medicine and Medical Biotechnologies, University of Naples Federico I, Via S.Pansini, 5 80131 Naples, Italy; CEINGE-Biotecnologie Avanzate s.c.ar.l, University of Naples ‘Federico II’, Via S.Pansini, 5 80131 Naples, Italy

**Keywords:** Epigenetics, Interleukin-4, Interleukin-5, Interleukin-10, Interferon-γ, Food allergy, Extensively hydrolyzed casein formula, Lactobacillus rhamnosus GG, Hypoallergenic formulae

## Abstract

**Background:**

Epigenetic changes in DNA methylation could regulate the expression of several allergy-related genes. We investigated whether tolerance acquisition in children with immunoglobulin E (IgE)-mediated cow’s milk allergy (CMA) is characterized by a specific DNA methylation profile of Th2 (IL-4, IL-5) and Th1 (IL-10, IFN-γ)-associated cytokine genes.

**Results:**

DNA methylation of CpGs in the promoting regions of genes from peripheral blood mononuclear cells and serum level of IL-4, IL-5, IL-10 and INF-γ were assessed in children with active IgE-mediated CMA (group 1), in children who acquired tolerance to cow’s milk proteins (group 2) and in healthy children (group 3). Forty children (24 boys, aged 3 to 18 months) were enrolled: 10 in group 1, 20 in group 2, and 10 in the control group. The DNA methylation profiles clearly separated active CMA patients from healthy controls. We observed an opposite pattern comparing subjects with active IgE-mediated CMA with healthy controls and group 2 children who outgrew CMA. The IL-4 and IL-5 DNA methylation was significantly lower, and IL-10 and INF-γ DNA methylation was higher in active IgE-mediated CMA patients. Gene promoter DNA methylation rates of all cytokines and respective serum levels were strongly correlated. Formula selection significantly influenced cytokine DNA methylation profiles in group 2.

**Conclusions:**

Tolerance acquisition in children with IgE-mediated CMA is characterized by a distinct Th1 and Th2 cytokine gene DNA methylation pattern. These results suggest that DNA methylation may be a target for CMA prevention and treatment.

## Background

Cow’s milk allergy (CMA) is one of the most common food allergies (FA) in early childhood, with an estimated incidence ranging between 2% and 3% [1]*.* Evidence suggests that the natural history of CMA is changing, with an increasing persistence until later ages [2]. It has been demonstrated that immunoglobulin E (IgE)-mediated CMA could be the first manifestation of the so-called ‘atopic march’, characterized by the occurrence of other allergic disorders in the years after the onset of CMA [3]. The pathogenetic basis of IgE-mediated CMA is not completely understood. Critical to the development of IgE-mediated chronic allergic inflammation is an increased production of Th2 cytokines, such as interleukins IL-4 and IL-5, which are not adequately counter-regulated by Th1 cytokines, such as interferon gamma (IFN-γ) and IL-10 [4]. We previously found an opposite response profile to these cytokines in freshly isolated peripheral blood mononuclear cells (PBMCs) between children with active IgE-mediated CMA and children who outgrew CMA [5]. Evidence suggests that CMA is mediated by a combination of genetic and environmental risk factors. A key mechanism that governs how environmental exposures modulate gene expression is DNA methylation of the cytosine of CpG dinucleotides in the gene-promoter regions, a well characterized epigenetic mechanism [6]. Therefore, the mechanism by which epigenetics contributes to CMA is a critical issue [7]. Epigenetics is the study of heritable changes in transcriptional activity that are not caused by changes in DNA sequence [8,9]. Epigenetic marks, such as DNA methylation, seem to work with other components of the cellular regulatory machinery to control the level of genes expression, and several allergy-related genes have been found to be susceptible to epigenetic regulation, including genes important for T-effector pathways, namely IFN-γ and IL-4 [7,10]. Preliminary data showed that epigenetic modifications at the Th2 locus could be responsible for the establishment, maintenance, and inheritance of the Th2 phenotype, while altered epigenetic regulation is potentially important for the exaggerated Th2 response seen in atopic diseases [11]. Decreased DNA methylation and an increased association with activating histone marks conjointly establish and maintain the euchromatin structure at the Th2 locus of Th2 cells, thereby allowing recruitment of the transcriptional machinery to this region for a rapid and coordinated expression of the Th2-related cytokines [12-14]. Hypermethylation of IFN-γ has been positively correlated with pro-allergic IgE production [15]. As DNA methylation is a reversible mechanism and can be changed during disease course [16-19], we evaluated whether tolerance acquisition in children with IgE-mediated CMA is characterized by a specific DNA methylation profile of cytokine genes associated with Th2 (IL-4, IL-5) and Th1 (IL-10, IFN-γ) response.

## Results

### Study subjects

From 3 December 2012 to 30 April 2014, 13 consecutive children with a recent evidence of IgE-mediated CMA were evaluated. All these patients presented with suspected CMA-related signs and symptoms within 5 weeks of study enrollment. Three were excluded because a negative oral food challenge. The ten remaining patients (group 1) were positive at the double blind placebo controlled food challenge (DBPCFC) and reacted between at doses 3 and 10 ml. During the same period, 20 subjects with a previous diagnosis of IgE-mediated CMA, but with recent evidence of oral tolerance acquisition demonstrated by a negative DBPCFC were also enrolled in the study (group 2). All subjects were able to consume at least one full cup daily of cow’s milk without symptoms. Group 2 patients, received for a median of 384 days (IQR 7.75) one of the following substitutive formulae: extensively hydrolyzed casein formula (EHCF, *n* = 3); extensively hydrolyzed casein formula containing the probiotic *Lactobacillus rhamnosus* GG (EHCF + LGG, *n* = 10); hydrolyzed rice formula (RHF, *n* = 1); soy formula (SF, *n* = 3); amino acid-based formula (AAF, *n* = 3). During the same period, ten healthy children were evaluated and their parents gave informed consent to the study. The main demographic and clinical characteristics of the study population are detailed in Table 1.

**Table 1:** **Main demographic and clinical characteristics of the study population**

	**Subjects with CMA at diagnosis**	**Subjects outgrown CMA**	**Healthy subjects**
	**Group 1**	**Group 2**	**Group 3**
*N*	10	20	10
Male, *n* (%)	7 (70)	13 (65)	4 (40)
Age, m (SD)	5.5 (0.7)	16.9 (0.9)	9 (4)
Body weight, kg (SD)	7.385 (964.7)	12.160 (720.9)	8.075 (3193.5)
Spontaneous delivery, *n* (%)	5 (50)	5 (25)	4 (40)
Breastfeeding, ≤8 weeks, *n* (%)	10 (100)	20 (100)	10 (100)
Symptoms at the CMA onset			
Gastrointestinal, *n* (%)	4 (40)	8 (40)	-
Cutaneous, *n* (%)	8 (80)	15 (75)	-
Respiratory, *n* (%)	1 (10)	6 (30)	-
Total serum IgE, kU/l (SD)	260.6 (230.9)	193.1 (209.5)	0.2 (0.1)
nBos d 4, kUA/l (SD)	6 (11.2)	1.9 (3)	-
nBos d 5, kUA/l (SD)	4.5 (7.1)	2 (3)	-
nBos d 6, kUA/l (SD)	5.4 (8.7)	1.4 (2.5)	-
nBos d 8, kUA/l (SD)	22.7 (39.2)	0.9 (1.9)	-
Lactoferrin, kUA/l (SD)	2 (6.1)	0	-

### Cytokines DNA methylation profiles

**Figure 1:**
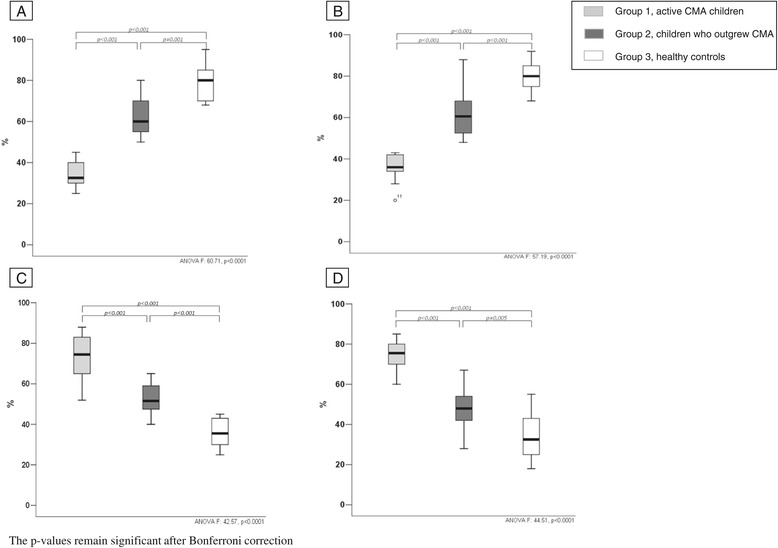
**DNA methylation rate of the IL-4, IL-5, IL-10, and INF-γ genes (A, B, C, D, respectively) observed in the study population.** The rate of promoter gene methylation of IL-4 and IL-5 was lower and IL-10 and IFN-γ was higher in group 1 than group 3. Group 2 presented a methylation rate similar to that observed in group 3. (group 3 *vs* group 1: coefficient −0.151, SE 0.058; *P* < 0.0001; group 1 *vs* group 2: coefficient 0.542, SE 0.2518; *P* < 0.0001).

Figure 1 shows the promoter DNA methylation profiles of IL-4, IL-5, IL-10, and INF-γ from PBMCs in the three study groups. The DNA methylation profiles clearly separated active CMA patients from healthy controls. We observed an opposite pattern between children with active IgE-mediated CMA and healthy controls. The DNA methylation of IL-4 and IL-5 was lower and of IL-10 and IFN-γ was higher in active CMA patients than in healthy controls (Figure 1A, B, C, D). DNA methylation analysis of these cytokine genes clearly also separated CMA patients by disease-state. Subjects with recent evidence of oral tolerance acquisition presented a different methylation profile if compared to active CMA patients. This profile was similar but not identical to that observed in healthy controls (Figure 1A, B, C, D). The backward stepwise Firth regression analysis showed that the best predictor of active CMA was the IL-5 methylation rate (healthy controls *vs* active CMA: coefficient −0.151, SE 0.058; *P* < 0.0001; active CMA *vs* patients who outgrew CMA: coefficient 0.5419, SE 0.2518; *P* < 0.0001).

**Figure 2:**
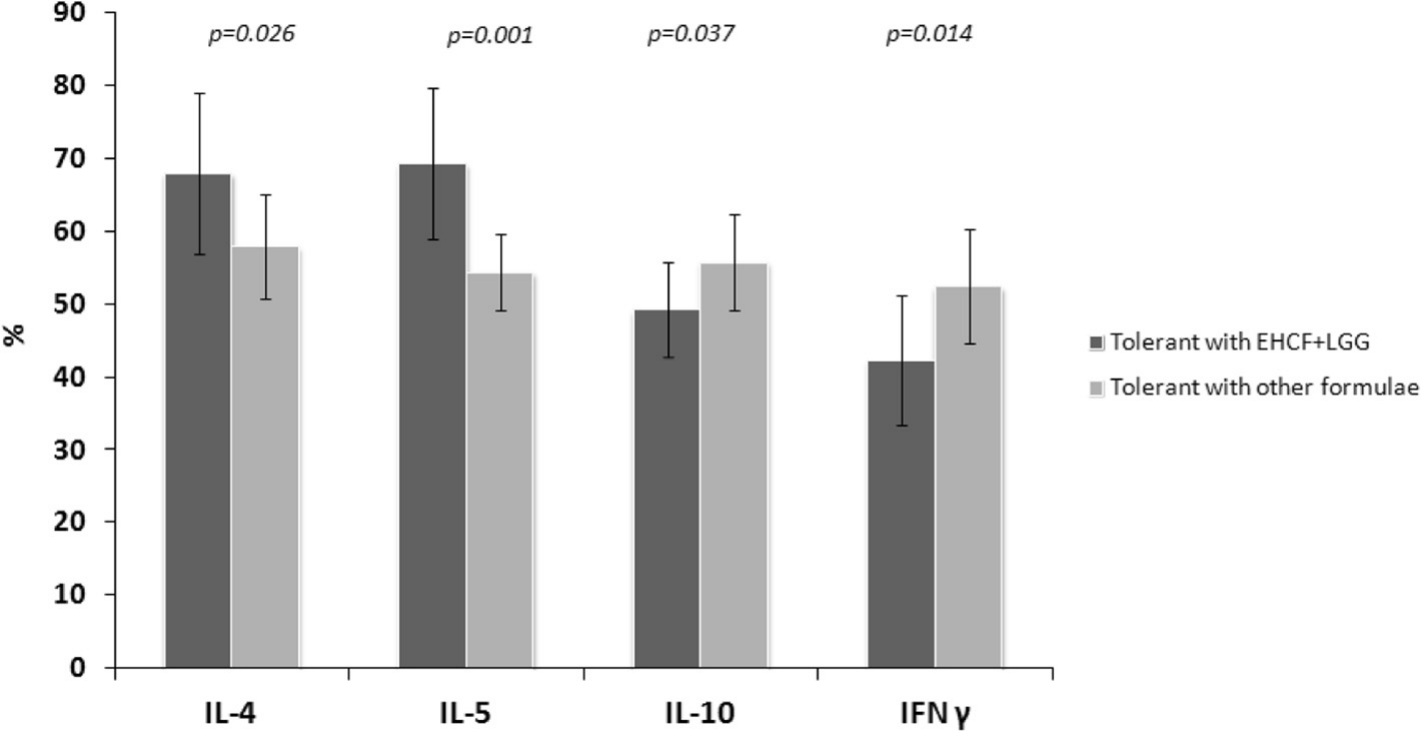
**Different DNA methylation rates of the IL-4, IL-5, IL-10, INF-γ genes in children who outgrew CMA receiving different dietary treatment.** Children, who outgrew CMA after EHCF + LGG treatment, presented a different cytokines methylation profile compared to children treated with other formulae.

We analyzed, through a linear regression analysis, potential factors (age, sex, serum IgE, specific serum IgE anti-epitopes, symptoms, formulae) able to influence IL-5 DNA methylation rate in subjects who outgrew CMA. The variable that most affected the DNA methylation rate of cytokines was the use of the EHCF + LGG formula (coefficient −17.761, SE 3.86, *P* = 0.004). Accordingly, patients who outgrew CMA after EHCF + LGG treatment presented a different cytokine methylation pattern compared to children treated with other formulae (Figure 2).

### Cytokine serum levels

**Figure 3:**
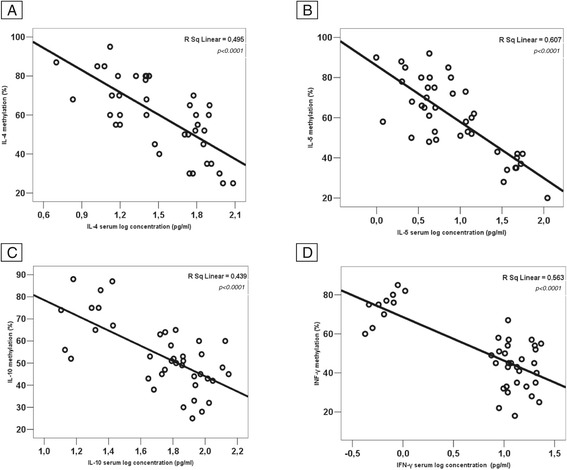
**Correlation between IL-4, IL-5, IL-10, and INF-γ gene DNA methylation rate and cytokine respective serum levels observed in groups 1, 2, and 3 (A-D).** There is a negative and significant correlation between the DNA methylation rate and the respective serum concentration for all cytokines.

**Figure 4:**
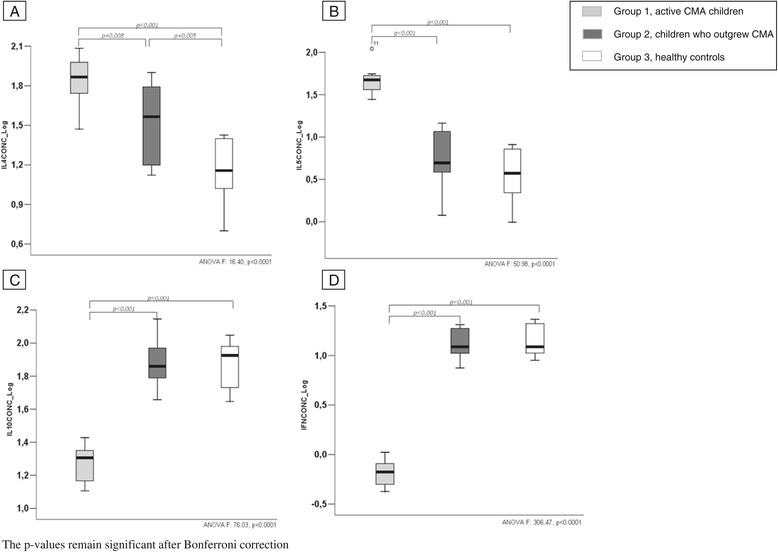
**Serum concentration of IL-4, IL-5, IL-10, and INF-γ (pg/ml) observed in groups 1, 2, and 3 of the study population (A-D).** Group 1 had showed significantly higher IL-4 and IL-5 and significantly lower IL-10 and INF-γ serum levels compared to group 3. Group 2 presented different levels of all cytokines compared to group 1 and a similar level to that observed in group 3.

We evaluated whether DNA methylation in the promoter region negatively correlated with the serum concentration of IL-4, IL-5, IL-10, and INF-γ of the three study groups. The DNA methylation rate of all cytokines significantly correlates with the respective serum concentration (Figure 3A, B, C, D). Active IgE-mediated CMA patients showed significantly higher IL-4 and IL-5 and significantly lower IL-10 and INF-γ serum levels compared to healthy controls (Figure 4A, B, C, D). Subjects with a recent evidence of oral tolerance acquisition presented a different profile compared to active CMA patients. This profile was similar to that observed in healthy controls (Figure 4).

## Discussion

We found that oral tolerance acquisition in children with IgE-mediated CMA is characterized by an epigenetic regulation involving Th1 and Th2 cytokine genes. The rate of DNA methylation in Th1 and Th2 cytokine gene promoters in PBMCs differed significantly between active CMA children and children who outgrew CMA. The two groups showed an inverse DNA methylation pattern of the IL-4, IL-5, IL-10, and IFN-γ genes. Moreover, a strong correlation between gene promoter DNA methylation rates of all cytokines was observed with the respective serum levels. The link between DNA methylation and gene expression has been explored in *in vitro* studies [20,21]. In a study of CD4^+^T lymphocytes of patients with asthma, stimulated with allergens, there was a significant correlation between the degree of methylation and IL-4 concentration [22]. Lovinsky-Desir *et al*. found a negative correlation between the DNA methylation rate of the IFN-γ promoter in CD4 + T lymphocytes and the relative IFN-γ gene expression in a cohort of children and adults with allergic asthma [23]; a similar negative correlation between IFN-γ DNA methylation and gene expression of T effector cells was reported by Kohli *et al*. in children exposed to secondhand smoke and air pollution [24]. Our study is the first to compare methylation status of Th1 and Th2 cytokine genes in children affected by IgE-mediated CMA at different disease stage. Martino *et al*. retrospectively examined genome-wide DNA methylation profiles in CD4^+^T cells from a birth cohort of children with IgE-mediated food allergy and age-matched non-allergic controls at birth and at age 12 months [25]. They found dysregulation of DNA methylation of MAPK signaling-associated genes during early CD4^+^T cell development. They suggested that this may contribute to a suboptimal T-lymphocyte response in early childhood potentially associated to the development of food allergy. The use of epigenetic biomarkers, mainly based on DNA methylation, is well established for the diagnosis and prognosis of tumors and has begun to be understood in other conditions such as autoimmune diseases [26]. The identification of DNA methylation marks in immune-function-related genes could encourage the development of new epigenetic-based-biomarkers that could have direct application in food allergy. DNA methylation level of selected Th1 and Th2 cytokines genes could be used as a potential biomarker in predicting tolerance acquisition*.* In this view, Syed *et al*. showed that the FOXP3 DNA methylation rate decreased during successful oral immunotherapy in patients with peanut allergy and remain stable when oral tolerance is achieved [27]. In accordance with these data, our results suggest that in IgE-mediated CMA children, the DNA methylation of the Th1 and Th2 cytokine genes is a dynamic process with substantial differences occurring during the disease course. The magnitude of these differences, if confirmed in other studies, could be the basis for a potential use of these epigenetic marks as biomarkers of disease status.

In our study, the DNA methylation rate of the Th1 and Th2 cytokine genes differed significantly between CMA children treated with EHCF containing the probiotic *Latobacillus rhamnosus* GG, who acquired oral tolerance, and CMA children treated with other formulae. Among the factors potentially influencing DNA methylation rate in subjects who outgrew CMA, the strongest variable was EHCF + LGG treatment. This finding supports the use of these epigenetic marks as potential therapeutic biomarkers for dietary intervention. Interestingly, similar epigenetic effects have been reported for the farm-derived bacteria *Acinetobacter lwoffli* F78 in a mouse model of ovalbumin-induced asthma [28].

The main limitations of our study are the relative small number of children and cytokines evaluated and the cross-sectional design. Longitudinal cohort studies in CMA children from the onset of symptoms to the acquisition of oral tolerance are advocated. Another limitation is the PBMCs-based epigenetic evaluations. Given the small amounts of blood, as expected in a study of very young children, cell sorting was not possible. Although CMA is a systemic condition, for which the study of methylation patterns in whole blood may be feasible [10,19], we cannot exclude the possibility that blood cell heterogeneity may have exerted a confounding effect.

## Conclusions

While genetics may play a role in CMA, it cannot explain the rapid epidemiological changes occurred in recent decades. There is a growing recognition that the epigenome is a key missing piece of CMA pathogenesis puzzle [10]. Our findings suggest that DNA methylation plays a role in tuning the course of CMA and dietary interference with epigenetic mechanisms might represent an innovative approach to target the development of oral tolerance.

## Methods

### Study population

We planned to enroll two categories of IgE-mediated CMA patients (aged 3 to 18 months) consecutively referred to our tertiary Center for Pediatric Food Allergy because of the necessity of an oral food challenge: *i.* patients with recent evidence of IgE-mediated CMA (‘active CMA patients’, group 1); and *ii*. patients with a previous diagnosis of IgE-mediated CMA, but with evidence of oral tolerance acquisition (‘subjects who outgrew CMA’, group 2).

In all patients, oral food challenge was requested to obtain diagnosis of CMA (group 1) or evidence of oral tolerance acquisition (group 2). The diagnosis of IgE-mediated CMA was made in all subjects according to the result of a (DBPCFC, namely, occurrence of typical symptoms within 2 h of the administration of the last dose) and the outcomes of diagnostic work-up, that is, a clear clinical history of typical IgE-mediated symptoms of CMA, and positive specific serum IgE against proteins and epitopes of cow’s milk [29]. Patients with a negative DBPCFC (group 2) were reassessed after 4 weeks to check the persistence of clinical tolerance. A venous blood sample (4 ml) was obtained in all patients at the end of the DBPCFC procedure for serological test and epigenetic analysis. Th1 and Th2 cytokine dosage and PBMCs sorting were carried out with the same blood sample. Subjects with other allergic disorders or food allergies, eosinophilic disorders of the gastrointestinal tract, food protein-induced enterocolitis syndrome, concomitant chronic systemic diseases, congenital cardiac defects, active tuberculosis, autoimmune diseases, immunodeficiency, chronic inflammatory bowel diseases, celiac disease, cystic fibrosis, metabolic diseases, lactose intolerance, malignancy, chronic pulmonary diseases, and malformations of the gastrointestinal tract were excluded. As controls, during the same study period, consecutive healthy children, not at risk for atopic disorders (that is, without any first degree family member affected by atopic disorders), visiting our Center because of minimal surgical procedures were also enrolled (group 3). A venous blood sample (4 ml) was collected from all these healthy subjects. These children were also assessed for the presence of food allergy and other allergic diseases at enrollment and 6 months after blood sampling by experienced pediatric allergists operating at the Center. The medical record of each child was recorded on a clinical chart. The study was approved by the Ethics Committee of the University of Naples ‘Federico II’ and was registered in Clinical Trials Protocol Registration System (ID number: NCT02062476).

### Oral food challenge

All food challenges were performed by DBPCFC, as previously described [30]. All oral food challenges took place at our Center on two separate days with a 1-week interval. Parents of children taking antihistamine were advised to withhold these medications for at least 72 h before and during the challenge. Randomization and preparation of the challenges were performed by experienced food allergy dieticians not directly involved in the procedures. Briefly, every 20 min, successive doses (0.1, 0.3, 1, 3, 10, 30, and 100 ml) of fresh pasteurized CM containing 3.5% fat or an amino acid-based formula were administered. Full emergency equipment and medications (epinephrine, antihistamines, and steroids) were available. The results were assessed simultaneously by three experienced pediatric allergists. Study subjects were scored for nine items divided into four main categories: *i.* General (lowered blood pressure plus tachycardia); *ii.* Skin (rash, urticaria/angioedema); *iii.* Gastrointestinal (nausea/repeated vomiting, crampy-like abdominal pain, diarrhea); and *iv*. Respiratory (sneezing/itching, nasal congestion/rhinorrhea, stridor deriving from upper airway obstruction or wheezing) on a 0- to 3-point scale (0, none; 1, light; 2, moderate; and 3, severe). If at least two of the three physicians independently scored any item at level 3, or 2 (or more) items at level 2, the test result was considered positive. Clinical symptoms occurring within 2 h of administering the highest dose were defined as ‘IgE-mediated reactions.’ The infants were observed for 2 h after the final dose, and then discharged. In the case of a positive DBPCFC, at any testing dose, the patient remained under observation until symptom resolution. If the patient did not show any symptom within the first 24 h, parents were advised to give one single feed of 100 ml of the tested formula (*verum* or placebo) everyday at home for 7 days. If any symptom occurred during this period, the patient returned to the outpatient clinic on the same day. After 7 days of *verum* or placebo administration, the patients were examined and the parents interviewed at the Center. To rule out a false-negative challenge result, parents were asked to contact the Center if any symptoms occurred in the 7 days after the DBPCFC procedures. The challenge was considered negative if the patient tolerated the entire challenge, including the observation period. Clinical tolerance acquisition was defined by the presence of a negative DBPCFC. The persistence of tolerance acquisition was checked after 4 weeks in all subjects with negative DBPCFC.

### Total IgE and specific IgE against proteins and epitopes of cow’s milk

Serum was obtained by centrifugation for 10 to 15 min. Serum was flash frozen and stored at −80°C until analysis. Serum total IgE and specific IgE against epitopes of cow’s milk were analyzed by enzymatic immunoassay (Phadia 100 ThermoFisher Scientific CAP system, Rodano Milano, Italy).

### Measurement of serum IL-4, IL-5, IL-10, and IFN-γ concentration

Serum samples were flash frozen and stored at −80°C until further analysis. The concentrations of IL-4 and IL-10 were measured with a Human IL4/IL10 Enzyme immunoassay kit (Boster Biological Technology, Ltd., Fremont, CA, USA) according to the type of stimulation and stimulant. Human IL-5 and IFN-γ ELISA, High Sensitivity (BioVendor, Brno, Czech Republic) were used to detect the IL-5 and IFN-γ serum concentrations. Absorbance was read at 450 nm. The minimum detection concentrations were 15.6 pg/ml for IL-4, 7.8 pg/ml for IL-5 and IL-10, and 0.78 pg/ml for IFN-γ.

### Leucocytes isolation, DNA extraction, and bisulfite modification

PBMCs were isolated from whole blood samples using the Ficoll-Paque (GE Healthcare, Uppsala, Sweden) methods. DNA was extracted using the DNA Extraction Kit (GE Healthcare). One microgram of extracted DNA was modified with sodium bisulfite to convert all unmethylated, but not methylated-cytosines to uracil. Bisulfite conversion was carried out using the EZ DNA Methylation Gold Kit (ZYMO Research Co., Orange, CA, USA), according to the manufacturer’s instructions. The converted DNA was stored at −70°C until used. Fully methylated and fully unmethylated DNA (Merck Millipore, Darmstadt, Germany) were used as controls for the optimization of the assay conditions and to calculate the percent of methylation (0% to 100%). The primer used for DNA methylation analysis of the promoter region of the IL-4, IL-5, IL-10, and IFN-γ genes was designed *in silico*, using MethPrimer (http://www.urogene.org/methprimer/) (Table 2).

**Table 2:** **The primer sequences used for high-resolution melting real-time PCR analysis**

**Primers**	**Sequence (5′-3′)**	**PCR product size (bp)**	**CpG sites (** ***n*** **)**
IL-4 F	AGGTTAGGAGATGGAGATTATTTTG	102	4
IL-4 R	TAAAACTACAAACACCTACCACCAC		
IL-5 F	AGGTTAGGAGATGGAGATTATTTTG	102	5
IL-5 R	TAAAACTACAAACACCTACCACCAC		
IFN-γ F	GAGTTTTGTTTTGTTATTTAGGTTGG	124	6
IFN-γ R	AATACCTATAATCCCAACTACTC		
IL-10 F	TTTATTGTGTTAATTAGGATGGTTT	154	7
IL-10 R	TAAACAAAAATCCAAAATACTTTTT		

### High-resolution melting real-time PCR and sequencing

Real-time PCR was performed with the LightCycler® 480 instrument (Roche Applied Science, Penzberg, Germany) using 96-well plates (Roche Applied Science). Extensive optimization experiments were performed in order to maximize PCR amplification efficiency, including PCR program parameters, Mg^2+^, primer and template concentrations. Sodium bisulfite-converted DNA (15 ng) was added to the PCR reaction mix, which consisted of high-resolution melting Master Mix (Roche Applied Science), 0.25 μM primers, and Mg^2+^ (2.5 mM). dH_2_O was used to supplement up to 20 μl. The real-time PCR protocol began with one cycle at 95°C for 10 min followed by 40 cycles of 95°C for 10 s, 61°C for 10 s, and 72°C for 10 s. Immediately after amplification, a re-annealing cycle consisting of 95°C for 1 min and a rapid cooling to 65°C for 1 min was introduced to prepare the melting curve acquisition step. Real-time fluorescence acquisition was set at the elongation step (72°C). Samples whose amplification begun late and sample whose relative fluorescence value on the raw melting-curve plot was low were not further processed. All PCR reactions were performed in triplicate for each sample. Melting data acquisition began at 69°C and ended in 95°C, using a ramp rate of 0.2°C/s. High-resolution melting analysis was also performed with the LightCycler® 480 instrument (Roche Applied Science) using 96-well plates (Roche Applied Science). Data processing included normalization and resulted on the normalized melting curves and the respective negative derivative of fluorescence over the temperature plots, using the LightCycler 480® gene scanning software. The settings for data collection were 50 fluorescence acquisition points per degree centigrade resulting on a ramp rate of 0.01°C/s. Comparison of the melting curve or the peaks of an unknown sample with those of the controls gave the semi-quantitative estimation for the methylation level of that sample. The results were confirmed by direct sequencing (Sanger method modified: ddNTPs labeled with four different fluorophores) and analyzed by capillary electrophoresis (analytical specificity and sensitivity of the test: >99%).

### Statistical analysis

The Kolmogorov-Smirnov test was used to determine whether variables (cytokine DNA methylation rates and serum levels of cytokines and IgE) were normally distributed. Serum levels of cytokines and IgE were not normally distributed (Kolmogorov-Smirnov test; *P* < 0.05) and thus were 10 log-transformed (Kolmogorov-Smirnov test; *P* > 0.05).

The *χ*2 test and Fisher’s exact test were used for categorical variables. To evaluate the differences among continuous variables, one-way ANOVA and the *t*-test were performed. To determine which groups in the sample differ, the Bonferroni correction was performed. Pearson’s correlation coefficient ‘*r*’ was used to evaluate the correlation between continuous variables.

Given the small sample size and relative issues of separability, a backward stepwise Firth’s bias reduced logistic regression was used to identify the best predictor of the CMA disease state [31]. This analysis was performed with the R package ‘l*ogistf*’. Linear regression was used to estimate the relationship between continuous variables. The level of significance for all statistical tests was two-sided, *P* < 0.05. All data were recorded in a dedicated database and analyzed by a statistician, blinded to the group assignment of patients, with SPSS software (SPSS Inc, version 21.0, Chicago, IL, USA).

## Abbreviations

AAF: amino acid-based formula
APT: atopy patch testing
CMA: cow’s milk allergy
DBPCFC: double blind placebo controlled food challenge
EHCF: extensively hydrolyzed casein formula
EHCF + LGG: extensively hydrolyzed casein formula plus *Lactobacillus* GG
IgE: immunoglobulin E
IL-10: interleukin-10
IL-4: interleukin-4
IL-5: interleukin-5
INF-γ: interferon-γ
LGG: *Lactobacillus rhamnosus* GG
PBMCs: peripheral blood mononuclear cells
RHF: hydrolyzed rice formula
SF: soy formula
SPT: skin prick testing
